# Common pitfalls in point-of-care ultrasound: a practical guide for emergency and critical care physicians

**DOI:** 10.1186/s13089-016-0052-x

**Published:** 2016-10-26

**Authors:** Pablo Blanco, Giovanni Volpicelli

**Affiliations:** 1 Intensive Care Unit, Hospital Dr. Emilio Ferreyra, 4801, 59 St., 7630 Necochea, Argentina; 2 Intensive Care Unit, Clínica Cruz Azul, 2651, 60 St., 7630 Necochea, Argentina; 3 Emergency Medicine, San Luigi Gonzaga University Hospital, 10043 Turin, Italy

**Keywords:** Ultrasonography, Point-of-care, Injuries, Critical care

## Abstract

**Background:**

Point-of-care ultrasonography (POCUS) is a widely used tool in emergency and critical care settings, useful in the decision-making process as well as in interventional guidance. While having an impressive diagnostic accuracy in the hands of highly skilled operators, inexperienced practitioners must be aware of some common misinterpretations that may lead to wrong decisions at the bedside.

**Objectives:**

This article provides a revision list of common POCUS misdiagnoses usually found in practice and offers useful tips to recognize and avoid them.

**Discussion:**

The following aspects were selected and reviewed: pericardial effusion vs. pleural vs. ascites vs. epicardial fat; right ventricle dilation in acute pulmonary embolism and inferior vena cava for volume status assessment in cardiac ultrasound; lung point and lung pulse misinterpretations and mirror artifacts vs. lung consolidations in lung ultrasound; peritoneal fluid vs. the stomach and a critical appraisal of gallbladder signs of acute cholecystitis in abdominal ultrasound; the rouleaux phenomenon vs. deep vein thrombosis or acute right strain in vascular ultrasound.

**Conclusions:**

Following some rules in technique and interpretation, and always integrating POCUS findings into the broader clinical context, most POCUS misdiagnosis can be avoided, and thus patients’ safety can be enhanced. Being aware of a list of common pitfalls may help to avoid misdiagnoses.

**Electronic supplementary material:**

The online version of this article (doi:10.1186/s13089-016-0052-x) contains supplementary material, which is available to authorized users.

## Introduction

Point-of-care ultrasonography (POCUS) is living a gold era in emergency and critical care medicine, because it is now widely recognized its usefulness in complementing the physical examination and serving as a safe interventional guidance at the bedside [1, 2]. Moreover, an ultrasonography-supported paradigm is gradually becoming a routine approach when caring for critically ill patients [3, 4]. However, it is important to highlight some rising concerns regarding provoked patients’ injuries that can result when POCUS is used inappropriately by novice or inexperienced practitioners. POCUS misdiagnoses due to inexperience may lead to errors in the treatment that may worsen patients’ outcomes or even be fatal. It is important to remind that before taking any action and being independent in the decision-making process, practitioners must be properly trained and well prepared to the possibility of misinterpretations. Nowadays, POCUS competences are covered in many curricula [1, 5–8] and included in most hands-on training programs, which represent the best way to teach properly POCUS and train new operators. Here, we report a revision of some common POCUS misdiagnoses that may be encountered in the common clinical practice. We will highlight the potential impact of these mistakes on patient outcome, and finally we will list several tips that may be of help for a correct interpretation of POCUS imaging.

### Cardiac ultrasound

#### Pericardial effusion versus other diagnosis

**Fig. 1:**
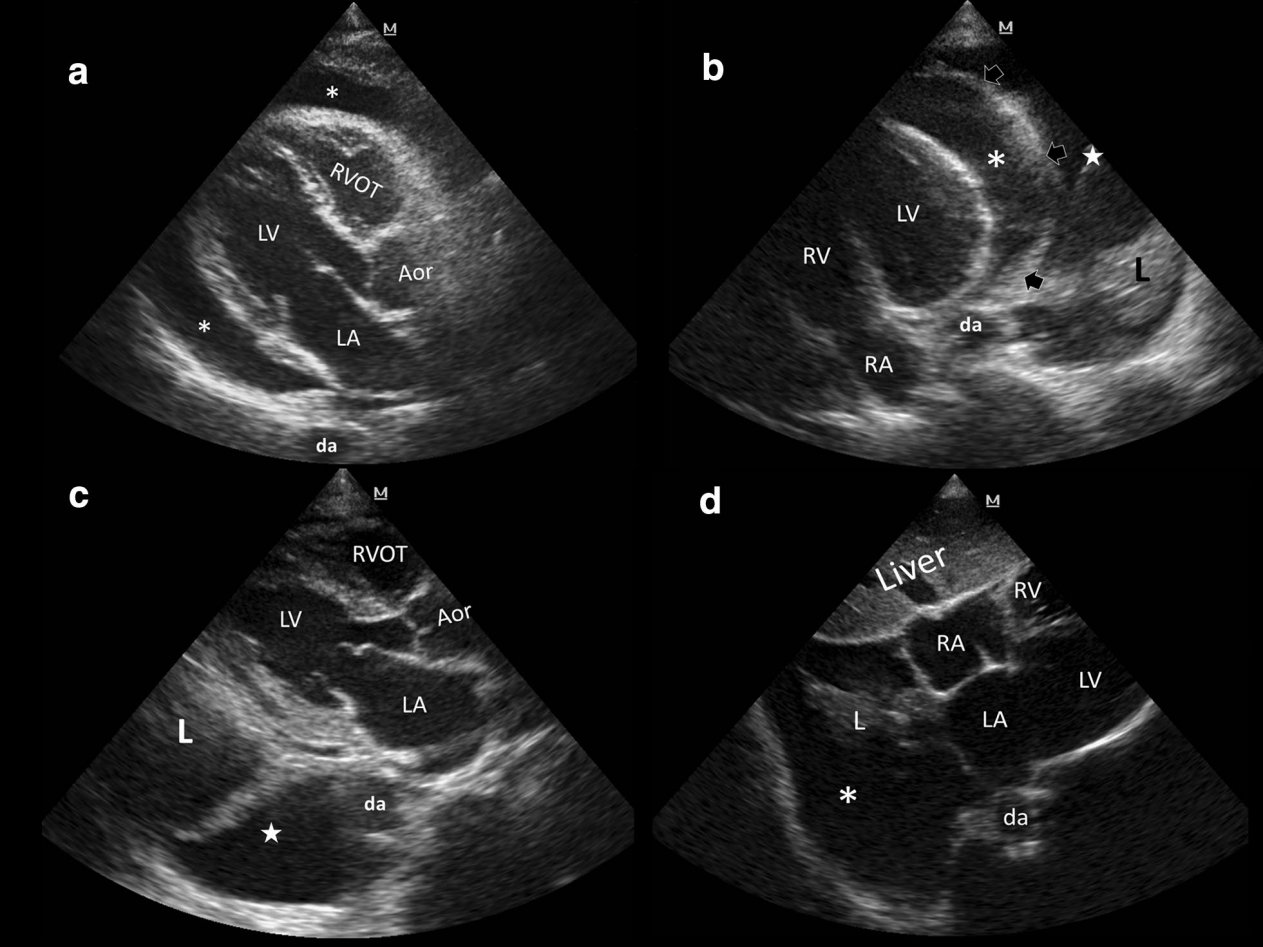
Pericardial versus pleural effusion. **a** Parasternal long axis view. Pericardial effusion (*asterisks*) tracks anterior to the descending aorta (da); *LV* left ventricle, *LA* left atrium, *RVOT* right ventricle outflow tract, *Aor* aortic root. **b** Apical 4-chamber view. Pericardial effusion (*asterisk*) tracks anterior to the descending aorta (da); pleural effusion (*white star*) tracks posterior and lateral to the da. *Arrows* pericardial delineation, *L* lung consolidation, *LV* left ventricle, *RV* right ventricle, *RA* right atrium. **c** Pleural effusion (*white star*) tracks posterior and lateral to the da; *L* lung consolidation, *LV* left ventricle, *LA* left atrium, *RVOT* right ventricle outflow tract, *Aor* aortic root. **d** Right pleural effusion (*asterisk*) extending into the bare area of the liver, besides the heart in subcostal 4-chamber view; *L* lung consolidation, *RA* right atrium, *RV* right ventricle, *LA* left atrium, *LV* left ventricle, *da* descending aorta
- PEF is always present in dependent segments (posterior wall, lateral wall, inferior wall).
- Checking diastolic collapse of cardiac chambers that is a sign of hemodynamically significant PEF: characteristically, the right ventricle outflow tract and the right atrium (RA) are the first parts of the cardiac chambers that collapse under the external pressure of PEF, followed next by the right ventricle (RV) with further increase in the pericardial pressure; diastolic collapse of the left atrium (LA) and left ventricle (LV) can be observed only in the most extreme cases [9, 10]. Right chambers will be fairly resistant to collapse in severe pulmonary hypertension, when abnormal high intracavitary pressure is opposed to the high intra-pericardial pressure [11]. Of note, diastolic collapse of both atriums in significant PEF occurs in ventricular systole (i.e., atrial diastole), and thus one can determine its presence when the atrioventricular valves are closed. This allow for distinction between diastolic collapses in PEF from the normal atrial systole which occurs when the atrioventricular valves are opened.
- Checking dilated (more than 2 cm diameter in long axis) and reduced or absent collapsibility (less than 20% collapse) of the inferior vena cava (tamponade) in spontaneously breathing patients [9, 10].

Pericardial effusion (PEF) is often found in critically ill patients and is defined by transthoracic echocardiography (TTE) as a diastolic fluid-filled space located within the two layers of the pericardium. Pericardial fluid is usually anechoic but sometimes can show a complex echotexture as, for instance, when it contains clots, pus or fibrin. PEF can be circumferential or regional. Even when circumferential, PEF not always is easy to visualize because it may distribute irregularly, and thus several TTE views are needed to demonstrate its presence and real amount. Magnitude and short time of collection are the two main characteristics that influence the possibility of tamponade. The most important tips to recall regarding PEF recognition at POCUS are:

- Tracking anterior to descending aorta in parasternal long axis and apical 4-chamber views (Fig. 1a, b) [9, 10].

In case of hypovolemic patients with small, under-filled cardiac chambers, even a mild PEF may be overestimated because the hyper contractile heart could simulate a diastolic collapse of the right chambers. In these cases, the observation of a depleted inferior vena cava (more than 50% or complete collapse during respiration) is a key finding, allowing to rule out a significant role of PEF in the hemodynamic instability.

The most common problematic differential diagnosis of PEF are represented by pleural effusion, peritoneal free-fluid (ascites) and epicardial and/or mediastinal fat. In addition, a large hiatal hernia, pericardial or other mediastinal cysts or left ventricle pseudoaneurysm may sometimes be hardly differentiated but more rarely.

Pleural effusion is defined as a fluid-filled space located within the pleural space. When not directly investigated by lung ultrasound but incidentally detected during TTE, pleural effusion is usually present in a significant amount. Characteristically, left sided pleural effusion appears posterior and lateral to the descending aorta in parasternal long axis and apical 4-chamber views (Fig. 1b, c) [9, 10]. In subcostal views, a right pleural effusion can also be visualized besides the right cardiac chambers, extending over the bare area of the liver (Fig. 1d). When observed in detail, a mobile and consolidated lung is visualized into the pleural fluid (Fig. 1b–d). Additionally, lung ultrasound in coronal views will also show this effusion.

**Fig. 2:**
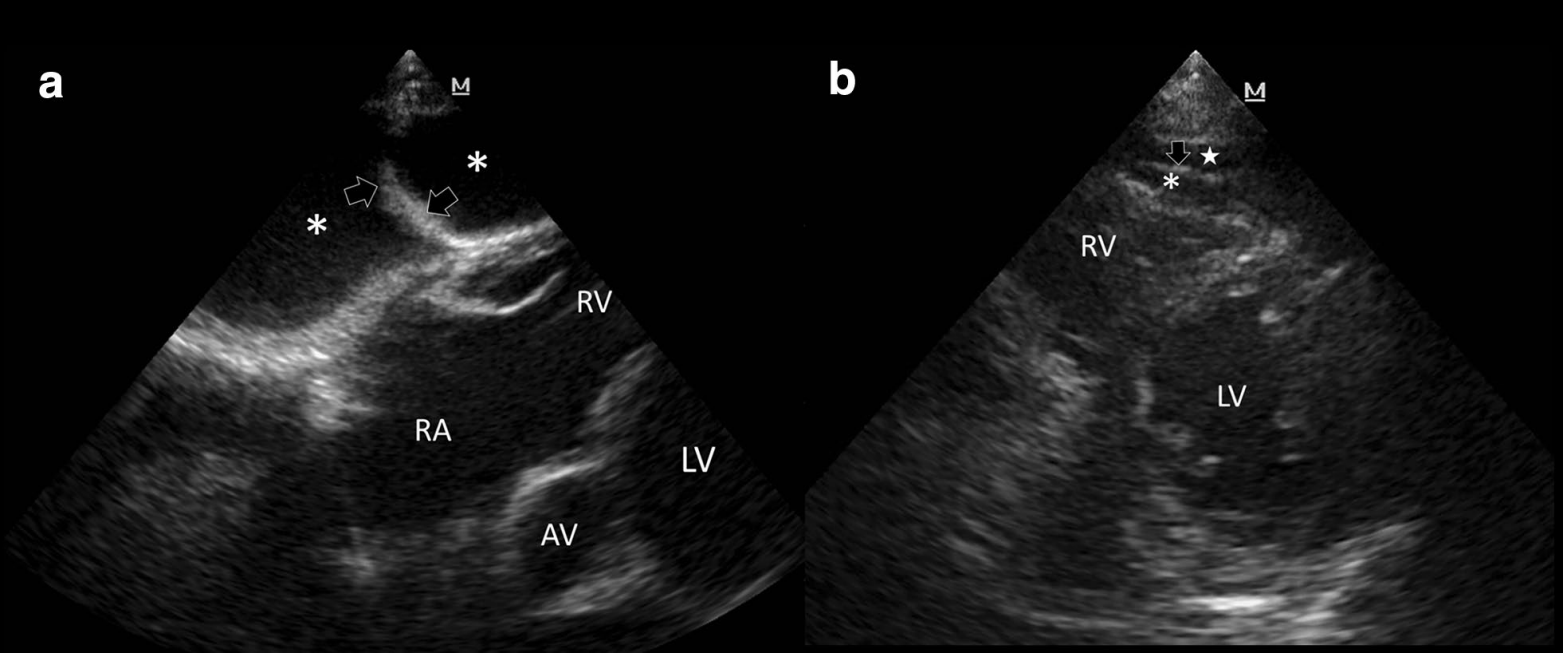
**a** Pericardial effusion versus ascites. Ascites (*asterisks*) is shown anterior to the heart in subcostal 4-chamber view. The falciform ligament (*arrows*) is depicted, confirming this effusion. *LV* left ventricle, *AV* aortic valve, *RV* right ventricle, *RA* right atrium. **b** Epicardial (*asterisk*) and mediastinal fat (*white star*), demonstrated in parasternal short-axis view, in systole; *LV* left ventricle, *RV* right ventricle, *arrow* pericardium

Ascites appears invariably in subcostal views, anterior to the right cardiac chambers. In these cases, the falciform ligament is observed within the fluid, which in addition with the visible diaphragmatic movement, allows to confirm the diagnosis (Fig. 2a) [12]. Moreover, extending the examination to the rest of the abdomen will easily demonstrate the presence of ascites to complete the diagnosis.

Epicardial fat is the adipose tissue accumulated between the visceral pericardium and the myocardium (i.e., epicardial region) [13, 14]. It is slightly isoechoic and is best observed in systole (obliterates completely in diastole) and exclusively in anterior regions. Thus, it is commonly visualized in parasternal long and short axis views (Fig. 2b) as well as in the subcostal 4-chamber view [13]. Mediastinal fat is located anterior to the pericardium and is best observed also in parasternal views (short and long axis) during systole (Fig. 2b) [13].

##### Error implication

An erroneously diagnosed PEF in the unstable patient may lead to undue pericardiocentesis with the strong possibility of cardiac chamber perforation, pericardial tamponade, and eventually, death.

##### How to avoid this error

It is essential to integrate the US findings in the clinical context and consider all the information on the patient condition, signs and symptoms; tamponade is a clinical diagnosis, which is supported by a compatible POCUS scan. Eventually, in doubtful cases it may be considered draining other effusions if these are well demonstrated and reevaluate thereafter the magnitude of a PEF. If PEF is still present but is not large and shows no hemodynamic consequences on the motility of the right cardiac chambers, drainage should be avoided unless an infectious PEF is suspected. In case of unusual regional location of PEF with intuitive difficulties in the procedure for draining, as it may be a posterior PEF, a surgical consult should be considered. In difficult cases, eventually, the correlation with other imaging methods (e.g., CT) is required.

#### Right ventricle dilation and acute pulmonary embolism

The diagnosis of right ventricle dilation is of paramount importance in the bedside diagnosis of pulmonary embolism in emergency situations and unstable patients. However, not always the dilation is clearly visible and many possibilities of error should be considered. The diagnosis of dilation basically relies on a comparison between the end-diastolic diameters of the RV and LV. A wrong and incomplete visualization of the cardiac chambers may lead to misdiagnosis. Moreover, the possibility of a chronic dilation not linked to acute right overload may induce further errors. This possibility may be encountered in case of evaluation of patients with chronic volume overload status (e.g., chronic significant tricuspid or pulmonary regurgitation) and chronic pressure overload conditions (e.g., chronic pulmonary hypertension) [15]. RV infarction is another condition coursing with RV dilation and impairment of RV function and sometimes hardly to differentiate from pulmonary embolism. In patients with cardiac arrest from any cause, ventricular chambers tend to equalize in short time, and thus applying RV dilation criteria may not be accurate for RV strain.

##### Error implication

A wrong diagnosis of RV dilation in the extreme emergency may lead to the erroneous conclusion of a state of acute right pressure overload (massive pulmonary embolism) that may induce undue thrombolysis or more invasive treatments (e.g., thrombectomy). On the other hand, missing the visualization of real RV dilation from an acute status may delay proper treatment and be harmful for the patient.

##### How to avoid this error

To diagnose correctly RV dilation, the two ventricles should be clearly and fully visualized, preferably in the apical 4-chamber view, and the two end-diastolic diameters taken beside at the level of the atrioventricular valve plane. When this view is not fully demonstrative, a conclusion should never be drawn. A ratio of at least 0.9 is indicative of dilated RV [16–19]. When other views are chosen for difficulty in obtaining a clear apical 4-chamber, different cut-offs should be applied (Table 1). Along with RV dilation, other signs of RV pressure overload may be considered, such as systolic interventricular septal flattening with systolic D-shaped RV (observed in short-axis views), impairment of RV systolic function (e.g., low TAPSE, RV free wall and RV inferior wall hypokinesia) as well as a RV-RA peak pressure gradient higher than 30 mmHg [15, 16, 19]. However, these additional signs are more laborious and need an advanced skill to be evaluated. Moreover, they are not sufficiently sensitive and specific when considered alone [15–17]. Finally, in cases of dilated RV, adjunctive signs of chronic dilation should be considered, like the thickness of the RV wall (superior to 5 mm), LV status, regional impairments, together with clinical history when available.

**Table 1:** Criteria for diagnosing right ventricle dilation at point-of-care bedside echocardiography (Adapted from [16]–[19])

Echocardiography view	Criteria for right ventricle dilation
4-Chamber apical view	Right/left ventricular end-diastolic diameter ratio >0.9
Parasternal long axis view	Right ventricular end-diastolic diameter >30 mm
Subcostal view	Right/left ventricular end-diastolic diameter ratio >0.7 or 0.9

For the evaluation of the dimension of the right ventricle the preferred view should be the apical 4-chamber. The other views should be used as second choice in case of doubtful measure or impossibility to obtain a clear image

RA thrombus in transit and thrombus located between both atriums in the foramen ovale are specific signs of pulmonary embolism, and thus should always be considered.

#### Inferior vena cava and fluid status

The inferior vena cava (IVC) is responsible for lower body systemic blood returning towards the heart. Thus, it is a common practice to consider that volemic status and/or fluid responsiveness may be in someway related to ultrasound measurement of the IVC diameter and its dynamics during the respiratory cycles, which allows a rough estimation of the right atrial pressure. While a depleted IVC is often observed in hypovolemic patients, typically the IVC is dilated in a hypervolemic condition with a less pronounced or null respiratory collapse [20]. However, some gray-zone conditions that alter this supposed linear relationship between the size of the vein and the actual volume status can be easily encountered and observed in practice. For instance, patients with high left ventricle filling pressures and normal IVC [21], potentially fluid responsive patients with physiologically dilated IVC [22], cases of acute right ventricular infarction, pericardial tamponade, acute massive pulmonary embolism, intraabdominal hypertension, asthma/COPD exacerbations and mechanically ventilated patients with positive pressures, all represent conditions where the IVC measurement may fail in indicating reliably the volume status. In addition, acute or chronic cor pulmonale, severe tricuspid regurgitation and pericardial constriction may show highly variable IVC dynamics, independent from the real volume status of the patient [23]. A further possibility of misinterpretation of the ultrasound study of the IVC may happen when the technique is not correctly applied. For a correct measure, the IVC should be visualized in long axis and the inner walls clearly imaged. In M-mode, the movement of the vessel under the probe may erroneously give the impression of a respiratory collapse that is not real. This must be checked observing simultaneously the M-mode with two dimensional imaging (Additional file 1: Video S1, Additional file 2: Video S2, Additional file 3: Video S3). Other phenomena observed is the inspiratory lateral translation of the IVC, producing a misalignment between the IVC and the US scanning plane, and thus simulating an inspiratory collapse [23]. These are common pitfalls that may trick even expert operators.

##### Error implications

These misinterpretations may lead to avoid fluid treatment and even using undue diuretics in patients who are volume depleted and potentially fluid responsive (e.g., in cases of patients mechanically ventilated, or in some emergencies like myocardial infarction of the right ventricle and obstructive causes of shock). On the other hand, errors in the IVC ultrasound may lead to erroneous administration of fluid challenges in patients with high left sided cardiac pressures or conditions of non-responsiveness to volume load, sometimes inducing pulmonary congestion, worsening hemodynamic status and even delaying proper administration of vasopressors and inotropes.

##### How to avoid this error

It is essential to integrate the IVC analysis with a comprehensive multi-organ ultrasound approach that should include a basic focused evaluation of the dimensions, ratio and function of the right and left cardiac chambers, a basic evaluation of the pulmonary congestion by assessing lung ultrasound for B lines, and of course, a full consideration of all the available clinical information. A correct interpretation of the volume status through the IVC assessment should always combine the respiratory variation of the IVC with its absolute size. In mechanically ventilated patients without inspiratory efforts and tidal volume >8 ml/kg (predicted body weight), IVC variation (distensibility index) distinguished fairly accurately the fluid responsive from non-responsive patients [23–25]. In patients with non-invasive ventilation, the interplay between the active respiratory efforts of the patient (negative pressure inducing an inspiratory flattening of the IVC) and the machine (positive pressure inducing an inspiratory engorgement of the IVC) has unpredictable effects on the inferior vena cava [23]. In spontaneously breathing patients with hypovolemia, a IVC collapsibility >42% may predict fluid responsiveness with high specificity (97%) [26]; however, normovolemic patients with large respiratory efforts may simulate this IVC dynamics [23]. Patients with shallow respiratory efforts may induce the opposite changes in the IVC collapsibility.

Other conditions affecting the transmural pressures between the thorax and the abdomen (e.g., asthma/COPD exacerbations, active expiratory efforts, patients with invasive mechanical ventilation and inspiratory efforts) may preclude the use of the IVC for volume assessment, as well as other situations that can induce high RA pressures (e.g., RV infarction, tamponade).

### Lung ultrasound

#### Lung sliding, lung point, B lines and lung pulse in the diagnosis of pneumothorax

The lung point is a fundamental ultrasonographic sign highly specific for confirming pneumothorax [27]. Two other equivalent signs are also highly specific for diagnosing pneumothorax [27]: the hydro-point [28] and the heart-point sign [29]. However, some normal physiological conditions can be misdiagnosed for false lung points [30, 31]. The requirement necessary to define the real lung point is an absent lung sliding with absent subpleural parenchymal ultrasound signals (B-lines or consolidations) and the lung point as the contact between the aforementioned region and the sliding lung (or pleural fluid as in hydro-pneumothorax) [32]. Indeed, some similar ultrasound patterns representing the interphases between the expanding lung and the diaphragm (Additional file 4: Video S4) and between the expanding lung and the heart (Additional file 5: Video S5), may be misdiagnosed for false lung points, especially when observed with high frequency probes, sometimes inducing the operator to wrong conclusions.

A complex situation can also be encountered in patients with pulmonary peripheral blebs. These patients commonly have structural lung parenchymal alterations that sometimes are at risk to develop a secondary pneumothorax in a background of chronic obstructive pulmonary disease [33]. Thus, differential diagnosis with pneumothorax may be crucial. In these cases, sometimes a regular lung sliding can be demonstrated in large peripheral blebs even if with some difficulty [34]. However, very often, the presence of pleural adhesions precludes the visualization of the respiratory movement of the lung and may induce erroneous interpretation. In stable patients absence of sliding should never be considered enough to finalize the diagnosis of pneumothorax. Especially, in patients with complex pulmonary disorders (large blebs and adhesions), a more detailed chest CT study may be required.

Pneumomediastinum may mimic a left sided pneumothorax, producing air artifacts during the chest ultrasound examination and even obscuring a regular parasternal and apical echocardiographic views. Correlation with chest X-ray or CT is usually required to confirm the diagnosis and exclude a pneumothorax [35].

While a false pattern of absence of sliding may contribute to misdiagnose pneumothorax, sometimes a respiratory or cardiac movement of the lung image at ultrasound may be misinterpreted for a false lung sliding (Additional file 6: Video S6). Even the beat of an intercostal artery or the thoracic internal artery in parasternal region may simulate a false lung pulse (Additional file 7: Video S7), thus inducing the operator to misdiagnose the absence of pneumothorax. Similarly, in complex cases of recurrence of pneumothorax after abrasion pleurodesis, the visualization of B lines due to multiple septa still connecting the lung to the chest wall may induce to erroneously rule out the condition (Additional file 8: Video S8) [36].

##### Error implications

The misdiagnosis of the absence of sliding or a false lung point may lead to an unnecessary pleural drainage. Furthermore, in doubtful cases, higher costs and irradiation exposure may arise if other unnecessary diagnostic imaging methods are ordered. In case of false sliding, false pulse or multiple septa, the diagnosis of pneumothorax may be dangerously delayed or even missed.

##### How to avoid these errors

Use a set of criteria to define pneumothorax by combining the four basic signs: abolished lung sliding, absent parenchymal signs (B lines and consolidations), absent lung pulse and look for the lung point for final confirmation in stable patients. Eventually, rule in a pneumothorax by chest CT if the patient is stable or correlate the ultrasound pattern with previous images, when available, in case of history of blebs or previous abrasion pleurodesis. In complicated cases, like secondary pneumothorax, recurrences, post-procedural pneumothorax, and trauma, a complex pattern should always be considered a strong possibility and induce the caring physician to carefully scan the whole chest to improve the accuracy of lung ultrasound.

#### Mirror artifact vs. lung consolidation

**Fig. 3:**
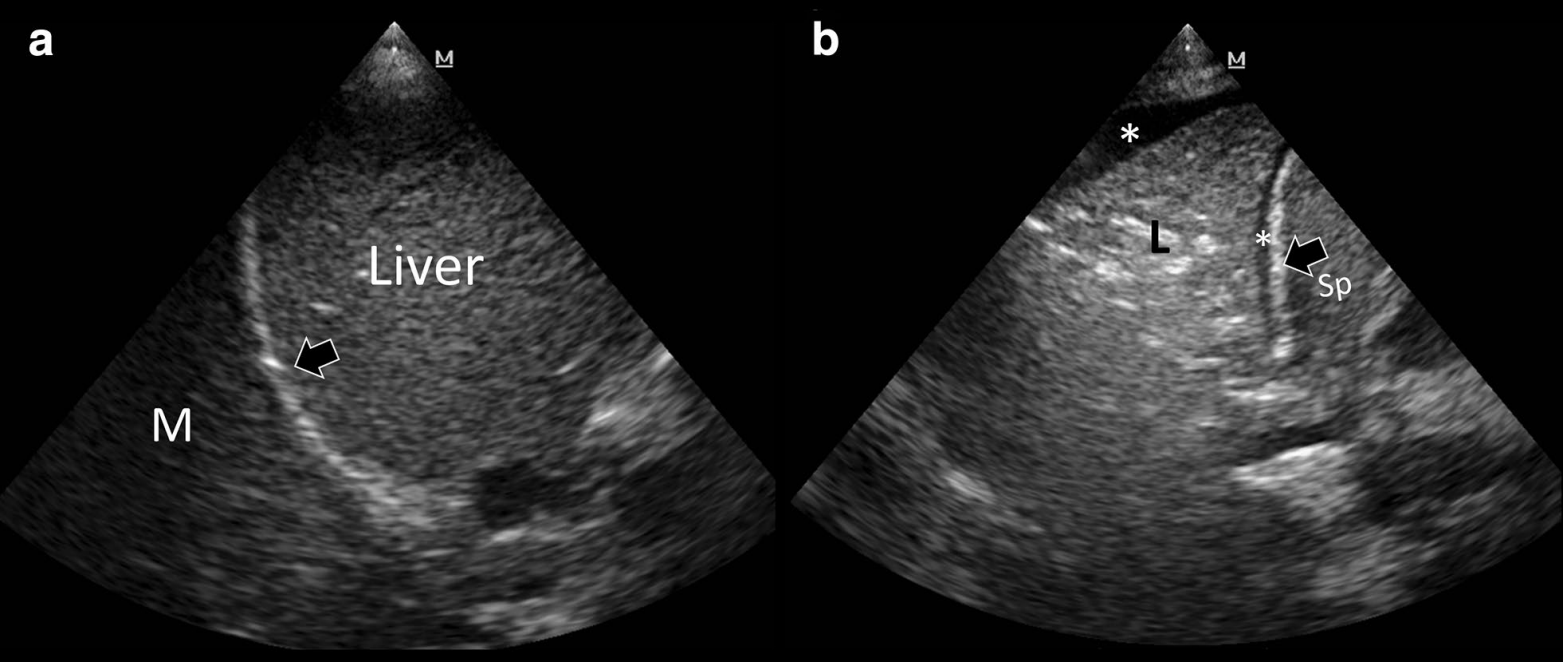
**a** Mirror image artifact (M), showing a similar tissue-like pattern in comparison with the liver; *arrow*: diaphragm. **b** Lung consolidation (L). Distinctively, it is shown a tissue-like pattern, sonographic air bronchogram and pleural fluid (*asterisks*); *arrow* diaphragm, *Sp* spleen

Mirror artifacts consist in the repetition of a false image resulting from the ultrasound beam hitting against a highly reflective surface [37], such as the diaphragm. Most frequent in the right side, this artifact can simulate a lung consolidation by the repetition of the liver tissue pattern above the diaphragm (Fig. 3a).

##### Error implications

This misdiagnosis may lead to higher costs and adverse effects derived from indications of antibiotics as well as unneeded chest X-ray or CT.

##### How to avoid this error

Along with integration into the clinical picture, observing the same tissue pattern of the liver or spleen is usually enough to rule in this artifact. The presence of pleural effusion that invariably accompanies lung consolidations and sonographic air or fluid bronchograms (Fig. 3b) is never observed in mirror image artifacts, and thus may aid in differentiating real lung diseases.

### Abdominal ultrasound

#### Peritoneal fluid versus the stomach

**Fig. 4:**
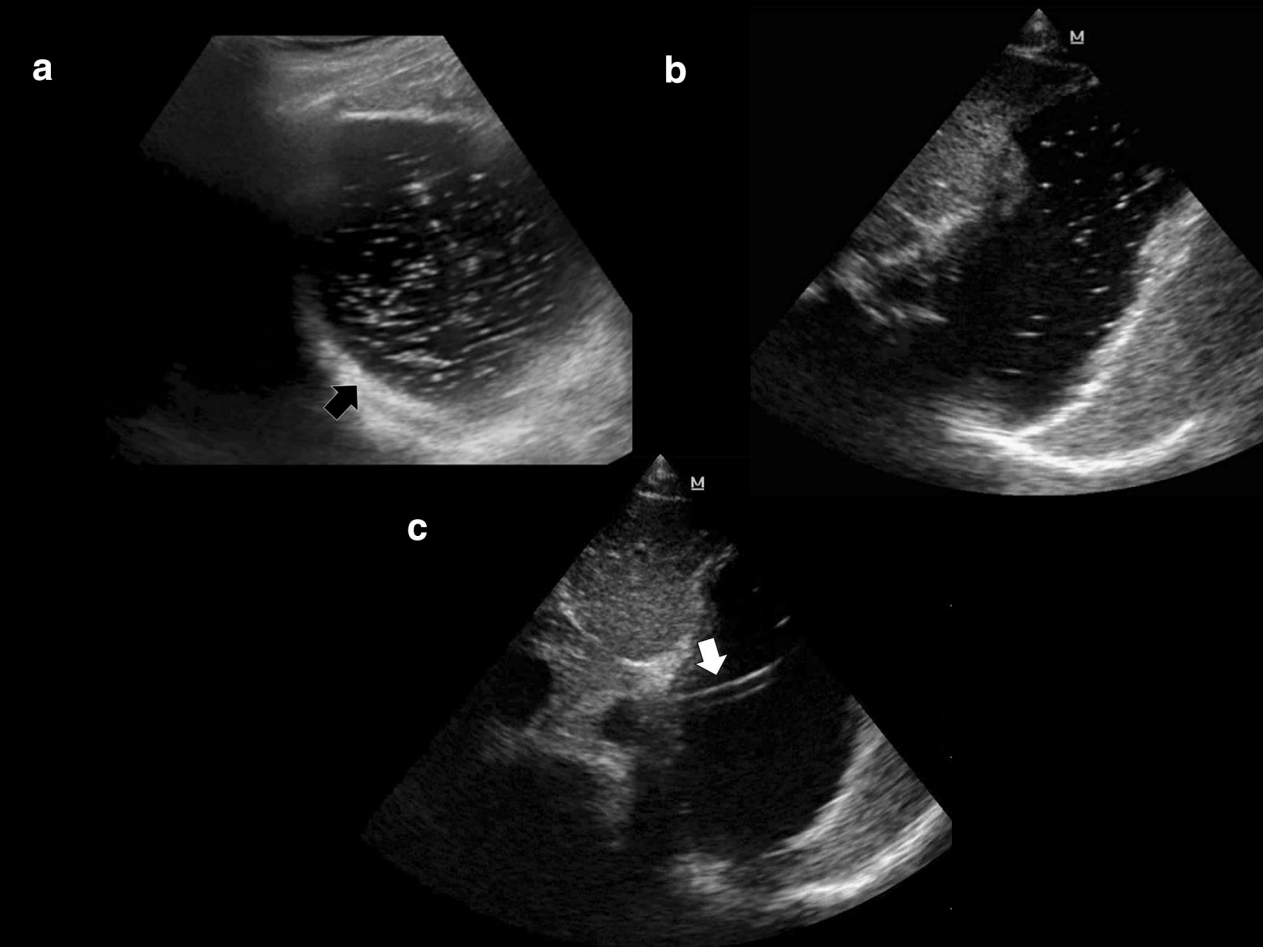
**a** Stomach observed from the left coronal view. Note the fluid content and the echoes inside (air bubbles); *arrow* diaphragm. **b** Stomach observed from an epigastric view simulating a collection. **c** A gastric tube is observed confirming that it is the stomach (*white arrow*)

Peritoneal fluid recognition is recommended during the first evaluation of trauma patients [38] as well as in patients presenting with several abdominal complaints and shock [39, 40]. When the abdomen is evaluated in left coronal views, a full stomach appearing below the diaphragm may mimic a condition of peritoneal fluid (Fig. 4a). In other situations, the stomach may even appear in epigastric subcostal views and may simulate a fluid abdominal collection (Fig. 4b). Depending on gastric contents (fluid, air, food), the appearance of the stomach may vary, ranging from simple anechoic fluid up to a heterogeneous ultrasound pattern.

##### Error implications

A misdiagnosis of false peritoneal fluid may lead to undue ultrasound-guided puncture and subsequent intraabdominal organ damage (i.e., spleen, diaphragm and stomach perforation), and eventually involving the pleural space causing pneumothorax. Furthermore, higher costs and undue irradiation may arise from confirmatory CT studies. Eventually, this misinterpretation may lead to laparotomy, and thus adjunctive surgical risks.

##### How to avoid this error

First, it is important to consider the possibility of a full stomach since it is visible in most non-fasted patients as well as in patients with delayed gastrointestinal emptying, which is a common condition in the critically ill. In doubtful cases and when the patients are able to take oral fluids, a possibility for differential diagnosis consists in observing bubbles appearing after having asked to the patient to drink some water. In unconscious patients or in extreme emergency situations, such as in postoperative abdominal patients with sepsis of unknown origin or in trauma patients, the opposite may be done by using the nasogastric or orogastric tube and evaluating if the image persists after gastric drainage. Moreover, the gastric tube can be also visualized by ultrasound coming down to the stomach during the maneuver, confirming the location of the organ (Fig. 4c).

#### Distended gallbladder, thickened gallbladder walls and acute cholecystitis

Along with a compatible clinical picture, ultrasonographic criteria of acute cholecystitis are a distended gallbladder, thickened walls, biliary sludge and lithiasis (calculus type), pericholecystic fluid and sonographic Murphy’s sign [41, 42].

**Fig. 5:**
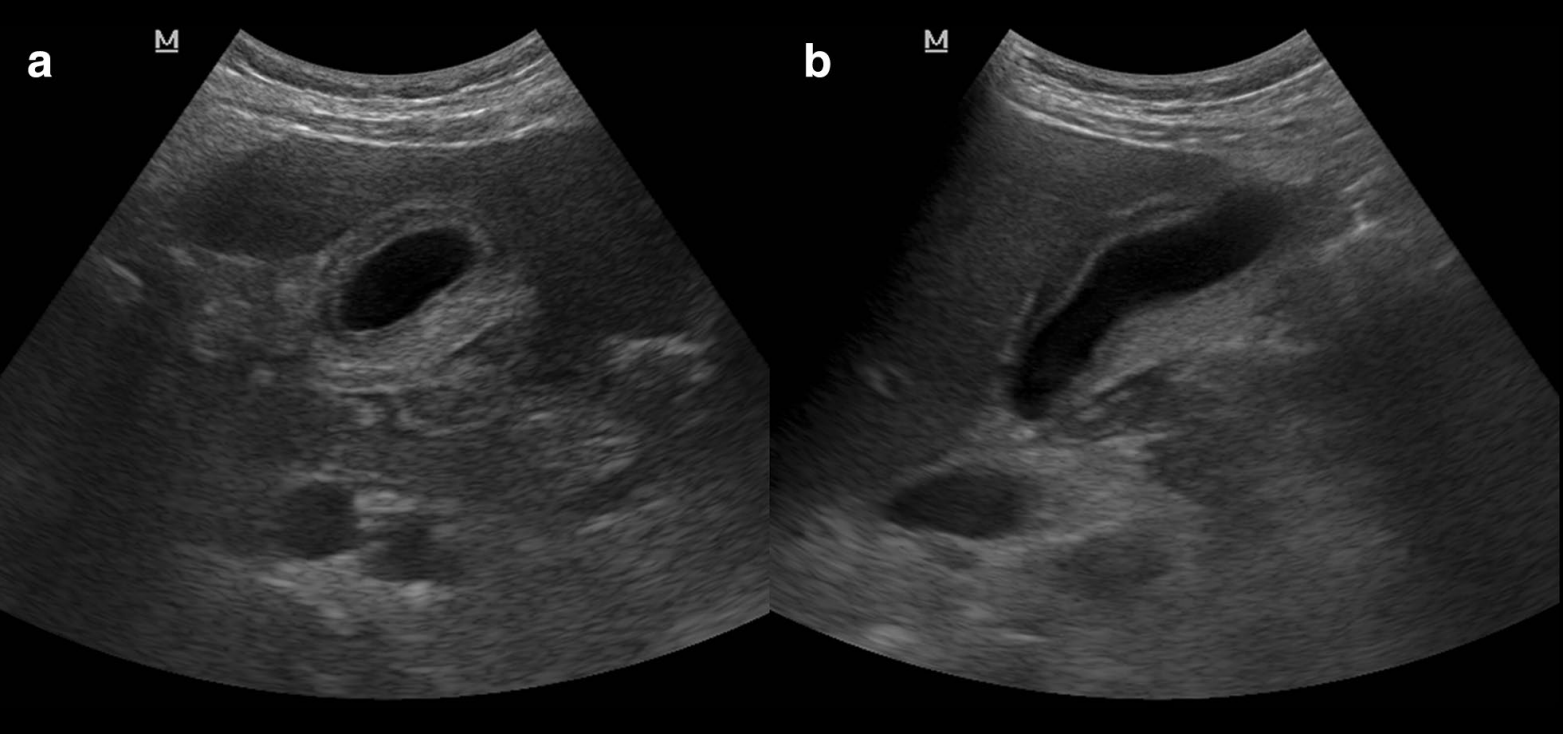
Gallbladder wall thickening in short (**a**) and long (**b**) axis in a patient with severe heart failure, hepatomegaly and ascites

Gallbladder distension and wall thickening, although important signs, are not specific for acute cholecystitis when considered alone (Tables 2, 3). For example, thickened gallbladder walls are commonly observed in patients coursing with generalized edemas such as those with cardiac failure (Fig. 5a, b), pre-eclampsia or renal failure. In non-fasted patients, the gallbladder is physiologically contracted, and thus may show thickened walls. In the presence of thickened walls, pericholecystic fluid and biliary sludge but the absence of clearly visible gallstones, acute cholecystitis during septic shock can be misdiagnosed [43].

**Table 2:** Causes of gallbladder wall thickening (Modified from [39])

Generalized edematous states	Inflammatory conditions	Miscellaneous	Neoplastic	Physiological
Congestive heart failure	Acute and chronic cholecystitis	Adenomyomatosis	Gallbladder adenocarcinoma	Non-fasting patient (contracted gallbladder)
End-stage cirrhosis	Cholangitis	Cystic vein varices	Metastases	
Renal failure	Acute hepatitis	Malabsorption		
Hypoalbuminemia	Pancreatitis			
Pre-eclampsia	Perforated duodenal ulcer

**Table 3:** Causes of gallbladder distension

Physiological	Obstruction
Fasting	Cystic duct (e.g., lithiasis) Common bile duct Pancreas

##### Error implications

This misdiagnosis may lead to higher costs and adverse effects derived from the indications of antibiotics, and eventually, to cholecystectomy or cholecystotomy, and thus unnecessary surgery risks.

##### How to avoid this error

It is essential to integrate ultrasound findings into the patient clinical context, always considering common underlying diseases resulting in morphological alterations of the gallbladder (e.g., cardiac failure). In the absence of clearly visible gallstones, the diagnosis of cholecystitis should always be confirmed by other methods [43].

### Vascular ultrasound

#### Rouleaux vs. deep vein thrombosis

**Fig. 6:**
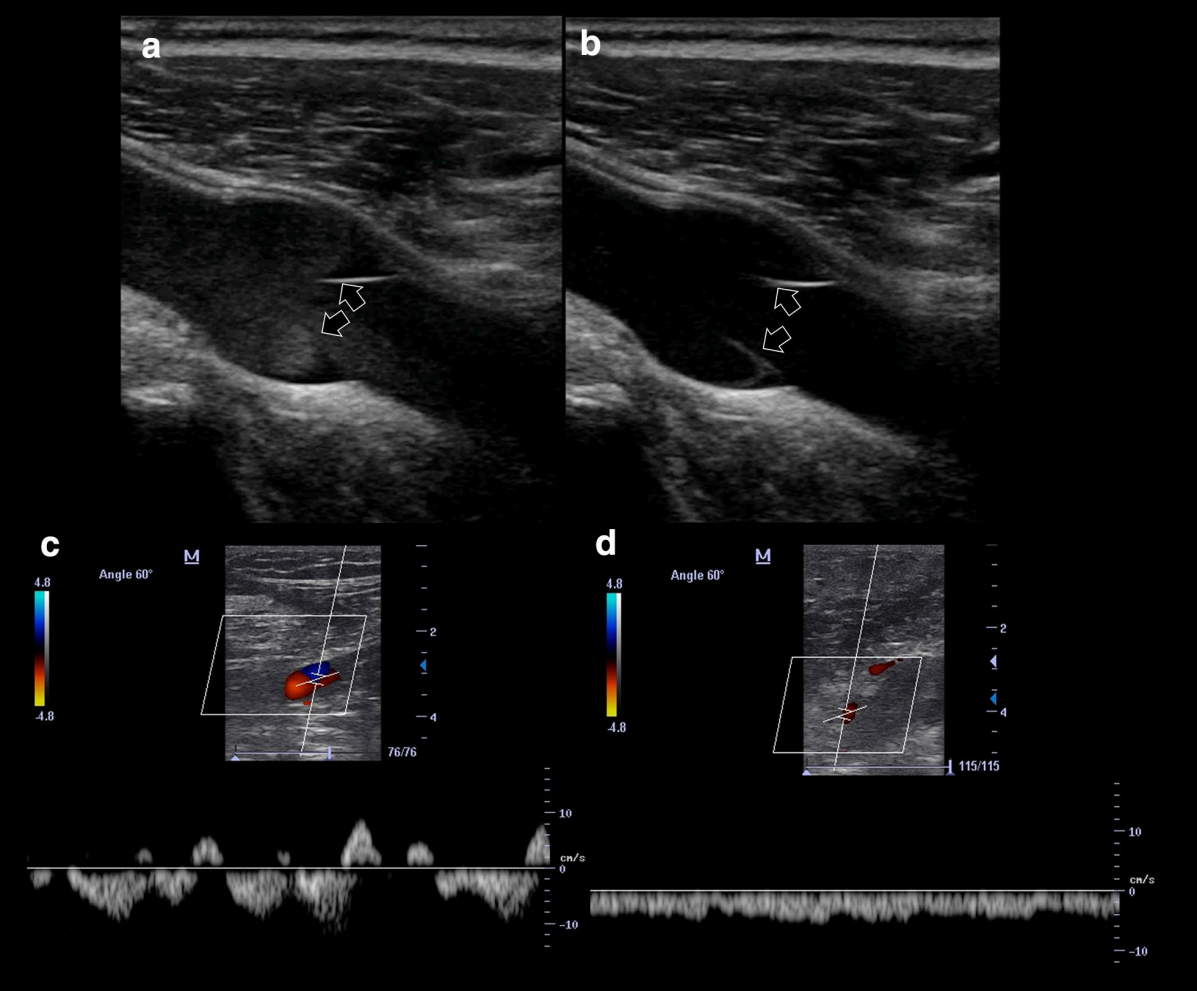
**a** Rouleaux formation over the venous valves (*arrows*). **b** After distal compression, the blood was squeezed and the rouleaux are finally cleared. *Arrows* venous valves. **c** Normal phasic venous waveform, indicating a non-stopping flow between the heart and the site of insonation. **d** Abnormal non-phasic venous flow, indicating a stop between the heart and the site of insonation

Most cases of deep vein thrombosis (DVT) are found in lower limbs, exposing patients to the risk of pulmonary embolism. A common differential diagnosis of DVT is the presence of “rouleaux formation” that is an accumulation of erythrocytes lying over the venous valves represented by spontaneously echogenic blood flow inside the vessel [44] (Fig. 6a; Additional file 3: Video S3). While rouleaux is a common finding and usually does not have a clinical impact, it is important to highlight that this can also be frequently observed when there is a proximal venous obstruction, and thus a more proximal DVT must be ruled out.

##### Error implications

A misdiagnosis of DVT only based on rouleaux may lead to higher costs and unnecessary side-effects (bleeding) derived from unnecessary patient anticoagulation. However, depending on the setting, the vessel observed and the patient condition, the rouleaux (or sludge sign) should sometimes induce to extend the observation to the possibility of proximal obstruction. The sludge sign in the inferior vena cava during cardiac arrest with electrical activity and hyperkinetic left cardiac chambers may be a sign of acute right overload [45]. The same sign in the femoral vein, corroborated by asymmetric femoral veins waveforms at color Doppler analysis, may be a sign of isolated iliac thrombus [45].

##### How to avoid this error

In contrast to a real condition of thrombosis, veins with rouleaux formation are compressible. Furthermore, distal compression allows to squeeze the blood and clear the rouleaux while thrombus cannot be released (Fig. 6b; Additional file 3: Video S3). To rule in or out a more proximal venous obstruction in the presence of rouleaux, the operator should look for direct visualization of a thrombus or examine compressibility of the proximal veins. In addition, the use of a spectral Doppler analysis to assess for respiratory flow variations of peripheral veins on both sides may be useful (Fig. 6c, d). More than a pattern of low intensity of the signal, especially, an asymmetric waveform obtained by comparing the two sides, indicates the possibility of a stop between the heart and the site of insonation and induces to extend the US venous study or use more advanced imaging for confirmation.

## Conclusions

There are some common misleading situations that critical care physicians may encounter in their sonography practice. Following some rules in technique and interpretation, the accuracy of POCUS can be improved and the possibility of dangerous misdiagnoses avoided. It is essential to remember that POCUS does not replace the clinical judgment, physical examination or common sense. POCUS must always be used in a holistic manner to improve the clinical accuracy and patient safety.

## Additional files

**Additional file 1: Video 1.** The inferior vena cava (IVC) in long axis in a patient with massive acute pulmonary embolism (Videos 1 to 3). The view of video 1 shows a significant venous collapse during respiration that is predictive of low venous pressure.

**Additional file 2: Video 2.** The inferior vena cava (IVC) in long axis in a patient with massive acute pulmonary embolism (Videos 1 to 3). In video 2, the M-mode documents the collapse of IVC during respiration.

**Additional file 3: Video 3.** The inferior vena cava (IVC) in long axis in a patient with massive acute pulmonary embolism (Videos 1 to 3). The same view (video 3) in long axis performed with the correct technique shows the absence of collapse in the obvious clinical condition of acute right strain. The difference is the correct displaying of the IVC in video 3, which is based on the constant visualization of the inner walls of the vessel.

**Additional file 4: Video 4.** Normal interphase between the expanding lung and the diaphragm. Note the ultrasonographic tissue pattern of the liver as well as the lung sliding.

**Additional file 5: Video 5.** Normal interphase between the heart and the expanding lung.

**Additional file 6: Video 6.** Lung ultrasound in a condition of pneumothorax. The movement of the muscles of the chest wall during the respiratory effort gives the impression of lung sliding. However, the real sliding should be observed against the chest wall and independent from the movement of the intercostal muscles.

**Additional file 7: Video 7.** Lung ultrasound in a condition of pneumothorax. The pulsing movement of the image is due to an intercostal artery visualized in long axis, giving the impression of a false pulsation of the lung. The real lung pulse should be independent from the movement of the chest wall.

**Additional file: Video 8.** A case of recurrence of pneumothorax after pleurodesis abrasion. The septa still connecting the lung to the chest wall explain the coexistence of B lines and the absence of sliding in a condition of pneumothorax.

**Additional file 9: Video 9.** Wash out of rouleaux formation after distal compression, ruling out thrombosis at the site of insonation.

## Abbreviations

POCUS: point-of-care ultrasonography
PEF: pericardial effusion
TTE: transthoracic echocardiography
RA: right atrium
RV: right ventricle
LV: left ventricle
LA: left atrium
IVC: inferior vena cava
CT: computerized tomography
DVT: deep vein thrombosis
